# A study of mobile phone use among patients with noncommunicable diseases in La Paz, Bolivia: implications for mHealth research and development

**DOI:** 10.1186/s12992-015-0115-y

**Published:** 2015-07-04

**Authors:** Kevin Kamis, Mary R. Janevic, Nicolle Marinec, Rachel Jantz, Helen Valverde, John D. Piette

**Affiliations:** University of Michigan School of Public Health, 1415 Washington Heights, Ann Arbor, MI 48104 USA; El Servicio Departamental de Salud (SEDES) La Paz, Calle Capitán Ravelo #2180, La Paz, Bolivia; Center for Managing Chronic Disease, University of Michigan School of Public Health, 1415 Washington Heights, Ann Arbor, MI 48109 USA

**Keywords:** Mobile health, Vulnerable populations, Latin America

## Abstract

**Background:**

While global momentum supporting mobile health (mHealth) research and development is increasing, it is imperative to assess the potential fit of mHealth programs in local settings. We describe the penetration of mobile technologies among Bolivian patients with noncommunicable diseases (NCDs) to inform research on mHealth interventions for the Andean region as well as low- and middle-income countries more generally.

**Methods:**

Five-hundred and fifty-nine NCD patients were identified from outpatient clinics affiliated with four hospitals in the cities of La Paz and El Alto. Respondents completed surveys about their use of standard mobile phones and smartphones. Respondents also provided information about their sociodemographic characteristics, health status, and access to care. We used descriptive statistics and logistic regression to understand the variation in mobile phone use across groups defined by patient characteristics associated with health service access and socioeconomic vulnerability.

**Results:**

Respondents were on average 52 years of age, 33 % had at most a sixth grade education, and 30 % spoke an indigenous language in their home. Eighty-six percent owned a mobile phone and 13 % owned a smartphone. Fifty-eight percent of mobile phone users sent or received a text message at least once a week. Some mobile phone owners reported connectivity problems, such as lacking mobile signal (9 %) or credit to make a call (17 %). Younger age, male gender, high health literacy, more years of education, and having fewer previously diagnosed NCDs were positively related to mobile phone ownership. Among mobile phone users, respondents with lower education and other indicators of vulnerability were less likely than their counterparts to report frequent usage of texting services.

**Conclusions:**

Mobile phones have high penetration among NCD patients in La Paz, Bolivia, including among those who are older, less educated, and who have other socioeconomic risk factors. Smartphone use is still relatively uncommon, even among patients who are younger and more educated. While certain patient characteristics such as age or education impact patients’ use of text messaging, mobile phone-based mHealth interventions are feasible strategies for increasing NCD patients’ access to self-management support between face-to-face clinical encounters.

## Introduction

Approximately 75 % of adults worldwide have access to a mobile phone [1]. The number of mobile phone subscriptions is increasing rapidly, and wireless communication tools are diffusing faster than any other communication technology [1, 2]. Three-quarters of the world’s seven billion mobile phone subscribers live in low- and middle- income countries (LMICs), making the developing world more *mobile* than the developed world [1, 3]. Some experts believe that the mobile phone provides more opportunities for development than any other invention in history [4].

One of the key economic and social sectors in which mobile phones are playing an increasingly important role is in healthcare service delivery and self-care support for patients, a field known as mobile health (mHealth) [5]. mHealth technologies encompass a variety of communication channels, including text messaging, video messaging, “live” voice calling, interactive voice response calls, and Internet-enabled smartphone apps [6]. mHealth service delivery models are attractive because they can serve a variety of functions critical for care management, including identifying patients at risk for developing acute health problems, providing frequent and tailored health education, and assisting patients with administrative tasks such as accessing health records and scheduling appointments [6–9]. Importantly, mHealth services can provide these functions when and where patients need them, extending the reach of health systems between face-to-face encounters [10].

mHealth strategies offer developing countries opportunities to combat a variety of high priority health problems such as HIV/AIDS and tuberculosis [11], and the United Nations, the World Bank, and the International Telecommunication Union agree that mHealth can be a vital tool for development [12]. Prior research studies suggest that mHealth services are feasible and acceptable in many low-resource areas including communities in Latin America [8, 13–18], and a number of studies have examined the impact of mHealth technologies on communicable diseases and maternal and child health in these settings [11, 19]. Still, most of the data on the use of mobile technology in the area of noncommunicable disease (NCD) and risk factor management have originated from developed countries, highlighting the need for more rigorous research in LMICs [11].

Greater research on the potential of mHealth in LMIC is particularly important given the large and rapidly increasing burden of NCDs globally. NCDs are a leading contributor to morbidity and mortality worldwide, and these conditions disproportionately affect less industrialized countries [11]. NCDs cause twice as many deaths as infectious diseases (including HIV/AIDS, tuberculosis, and malaria), maternal and perinatal conditions, and nutritional deficiencies combined [20]. Almost 80 % of deaths caused by NCDs occur in LMICs [20, 21]. In Latin America, more than 100 million adults are hypertensive, representing some of the highest rates of hypertension in the world [22]. In addition to physical health conditions such as diabetes, depression is a major contributor to NCD burden worldwide [23–25], and health systems in LMICs often lack the infrastructure and resources to address this burden. Given this epidemic, feasible and effective strategies for using mHealth could play a critical role in addressing the burden of NCDs in resource-constrained settings [11].

To that end, it is important to understand mobile phone distribution and use patterns among people living with NCDs so that mHealth strategies can be effectively designed to match local contexts [1, 26]. Unanswered questions remain about the extent to which mHealth services are feasible across locations and sociodemographic groups [27]. The current study addresses these issues for one important population in an understudied region, i.e., NCD patients seeking primary care in one of the poorest areas of the Andean region in Latin America – La Paz, Bolivia and its sister city, El Alto.

Specifically, we used survey data from NCD patients identified in ambulatory care clinics affiliated with four hospitals to examine factors influencing mobile phone ownership, use of phones with Internet connectivity (smartphones), patients’ ability to send and receive text messages, and barriers to mobile phone use. We focus only on mHealth technologies that could be implemented through patients’ own standard mobile phone or smartphone. By describing how demographic factors, health status, and health care access affect ownership and usage of mobile technologies in the Bolivian context, this study informs future mHealth research that includes NCD patients in that region as well as in similarly-challenged regions of Latin America.

## Methods

### Patient identification and recruitment

The study was approved by Human Subjects Committees at both the University of Michigan and the Universidad Católica Boliviana (UCB or Bolivian Catholic University). Between May and August 2013, as part of an ongoing series of projects aimed at establishing and testing a mHealth platform for NCD self-management support in Bolivia [28]. We administered face-to-face surveys at the time of primary care visits at three hospitals in La Paz, Bolivia and one hospital in the nearby city of El Alto. Potential participants were identified and approached in waiting rooms after they arrived for a non-urgent medical visit. To be eligible, respondents had to be at least 18 years of age and Spanish-speaking.

The sample of patients participating in the initial survey included 1144 adults; however, analyses presented here were limited to the subset of 620 who completed a more extensive survey module including questions about chronic illness management and cell phone use. This sample includes individuals with previously diagnosed NCDs in addition to those with probable and/or unmanaged depression and hypertension. Specifically, patients in this sample: (1) reported being told by a physician that they had one or more diagnosed chronic diseases including diabetes, hypertension, CVD (heart attack, blocked arteries, and/or stroke), depression, cancer, stroke, arthritis, respiratory disease (chronic obstructive pulmonary disease, asthma, and/or emphysema), and chronic back pain; or (2) had systolic blood pressures > 140 mmHg at the time of the visit, indicating potential hypertension; or (3) screened positive for depression on the Patient Health Questionnaire (PHQ) two-item depression screener [29]. Survey respondents received incentives in the form of personal hygiene materials (e.g. shampoo, toothpaste) and all completed written informed consent.

### Survey design and data collection

Surveys were administered by trained research assistants fluent in Spanish. Surveys used previously-validated translations of scales whenever possible (e.g., for measuring depressive symptoms) and the entire survey was reviewed and modified by native Spanish speakers in Bolivia prior to participant recruitment. The survey captured information about respondents’ sociodemographic characteristics, use of health services, and chronic disease diagnoses. Additional details of the survey methodology are presented elsewhere [28].

### Outcomes

Analyses focused on indicators of mobile phone access, use, and connectivity barriers. First, we examined the proportion of patients who reported owning a mobile phone, as well as the subset of phone users who owned a smartphone. Second, we examined patients’ use of text messaging within the subset of patients reporting mobile phone ownership. Specifically, we examined the odds of patients reporting whether they typically sent or received a text message at least once a week. Finally, we examined whether respondents with a mobile phone reported each of two common barriers to mobile phone use: frequently or always being unable to access a mobile phone signal, and frequently or always lacking mobile phone credit or “minutes” on their device.

### Predictors

Patients’ *sociodemographic characteristics* were self-reported and included: age, gender, indigenous ethnicity, and educational attainment. As a proxy for indigenous ethnicity (e.g. Aymara or Quechua), we identified patients who reported speaking one or more indigenous languages in their home at least some of the time (yes versus no). Patients were classified as having *low health literacy* if they reported that they were unable to read or had health literacy deficits as defined by two items from a commonly-used functional health literacy screener [30], i.e., frequently or always needing someone to help read papers from health center, or frequently or always having problems understanding written medical instructions. *Health status indicators* included: the widely used indicator of self-rated health (poor or fair vs. good, very good, or excellent) [31–33], the number of self-reported physician-diagnosed chronic conditions (0, 1, or ≥ 2), and a positive screen for depression using the Patient Health Questionnaire (PHQ-2) depression scale [29]. Patients were identified as having *cost-related barriers to health care access* if they responded yes to the question, “In the past year, did cost keep you from going to the clinic or hospital?” Patients’ *geographic access to care* was defined according to the time it typically took them to travel to clinic (0–29 min, 30–59 min, and ≥ 60 min).

### Analysis

Data were analyzed using SAS/STAT® software. All statistical tests were conducted using a 5 % significance level (α = 0.05). First, we examined the bivariate associations between (a) patients’ sociodemographic characteristics, health status, and access to care; and (b) owning any mobile phone and owning a smartphone. Among respondents with a mobile phone, we also examined bivariate associations between patient characteristics and frequency of text messaging usage and connectivity barriers. Multivariate logistic regression was used to assess variation in phone ownership and text messaging across patient groups controlling for covariates.

## Results

### Patient characteristics

The comprehensive survey was administered to a total of 620 individuals. After listwise deletion of cases with missing data on key variables, the final analytic sample size was 559 for models predicting phone ownership and 452 for those predicting text messaging. The majority of cases were excluded because of missing data on texting frequency (5.4 % missing) and years of education (3.5 % missing). All other variables had fewer than 5 % missing cases. Those excluded from this analysis were older, and significantly (p < 0.05) smaller proportions of those excluded were of high health-literacy (55 % of those excluded versus 69 % of the included sample) or owned a mobile phone (75 % of those excluded versus 86 % of those included). Texting ability was more common among mobile phone owners included in theses analyses than those excluded (76 % in the sample vs. 61 % excluded).

The majority of the 559 respondents were female (64 %), 10 % were less than 30 years of age, and 22 % were age 65 or older (Table 1). The mean age was 52 years (SD = 15.2). Almost one-third (30 %) of the participants spoke an indigenous language at home, 33 % had no more than 6 years of education, and 31 % were classified as having low health literacy. Most respondents (67 %) reported fair or poor health, and almost 42 % reported having two or more previously diagnosed NCDs. Many respondents (45 %) were identified as potentially having depression, according to their PHQ-2 score. Barriers to health care access were common, with 47 % reporting a cost-related access barrier and 26 % traveling at least an hour to their source of primary care.

**Table 1:** Sample Characteristics

	Total sample	Owns Mobile Phone*		Owns Smartphone	
	% (n)	%	*p*-value^§^	%	*p*-value^§^
Total	100 (559)	86.2		12.7	
Demographics					
Age			<0.01		<0.01
18–29	9.5 (53)	96.2		32.1	
30–49	32.0 (179)	91.1		16.2	
50–64	36.1 (202)	88.6		8.4	
65+	22.4 (125)	71.2		6.4	
Gender			<0.01		0.64
Male	36.3 (203)	91.6		11.8	
Female	63.7 (356)	83.1		13.2	
Indigenous language used at home			0.07		0.02
Yes	30.2 (169)	82.3		7.7	
No	69.8 (390)	88.0		14.9	
Health Literacy^a^			<0.01		<0.01
Low	30.9 (173)	72.3		4.6	
High	69.1 (386)	92.5		16.3	
Education in years			<0.01		<0.01
≤6	32.6 (182)	76.9		5.5	
7–12	38.1 (213)	87.3		8.9	
>12	29.3 (164)	95.1		25.6	
Health Status					
Self-Reported Health			0.81		0.02
G./V.G./Exc.^b^	32.6 (182)	85.7		17.6	
Poor or fair	67.4 (377)	86.5		10.3	
Number of Self-Reported Chronic Conditions			<0.01		0.03
0	23.3 (130)	93.9		16.9	
1	34.5 (193)	87.1		14.0	
≥2	42.2 (236)	81.4		9.3	
Depressed^c^			0.94		0.54
Yes	44.5 (249)	86.4		13.6	
No	55.5 (310)	86.1		11.9	
Health Care Access					
Cost-related access barrier^d^			0.43		0.01
Yes	46.5 (260)	85.0		8.9	
No	53.5 (299)	87.3		16.1	
Travel time to clinic in minutes			0.18		0.03
0–29	39.4 (220)	85.9		15.9	
30–59	34.5 (193)	87.6		12.4	
≥60	26.1 (146)	84.9		8.2	

All percentages are row-percentages. ^§^Result of Pearson Chi-squared Test for binary predictors, result of Cochran-Armitage test of trend for variables with more than two levels. *Shared mobile phone: n = 30 (6.3 %); ^a^Defined as not being able to read, frequently or always needing someone to help read papers from health center, frequently or always having problems understanding written medical instructions; ^b^Good/Very Good/Excellent; ^c^As measured by a score of 3 or more on the PHQ-2 questionnaire; ^d^“In the past year, did cost keep you from going to a clinic or hospital?”

### Access and use of mobile phone technology

Most participants (86 %) owned a mobile phone, and 13 % of all respondents owned a smartphone (Table 1). 58 % sent or received a text at least once a week (Table 2). Few respondents reported connectivity barriers; only 9 % reported frequently or always having difficulty obtaining a mobile signal, and 17 % reported frequently or always being without mobile phone credit to make a call (Table 2).

**Table 2:** Text Message Usage and Barriers to Mobile Phone Use Among Mobile Phone Owners

		Text Message Usage		Barriers to Mobile Phone Use			
	Total	Sends or Receives at least once a week		Frequently or Always Lacks Mobile Signal		Frequently or Always Lacks Mobile Phone Credit	
	% (n)	%	*p*-value^§^	%	*p*-value^§^	%	*p*-value^§^
Total	100 (452)	58.0		9.1*		16.5**	
Demographics							
Age			<0.01°		0.10°		<0.01
18–29	11.1 (50)	94.0		10.0		32.7	
30–49	35.2 (159)	74.2		11.3		18.9	
50–64	36.1 (163)	44.2		9.9		14.2	
65+	17.7 (80)	31.3		2.5		6.3	
Gender			0.06		0.76		0.12
Male	39.2 (177)	52.5		9.6		13.1	
Female	60.8 (275)	61.5		8.8		18.7	
Indigenous language used at home			<0.01		0.18		0.38
Yes	28.5 (129)	44.2		6.2		14.1	
No	71.5 (323)	63.5		10.3		17.5	
Health Literacy^a^			<0.01		0.65		0.46
Low	25.0 (113)	38.1		8.0		18.8	
High	75.0 (339)	64.6		9.4		15.7	
Education in years			<0.01		0.01		0.29
≤6	26.8 (121)	29.8		6.7		18.3	
7–12	39.2 (177)	56.5		5.7		17.6	
>12	34.1 (154)	81.8		14.9		13.7	
Health Status							
Self-Reported Health			0.01		0.19		0.84
G./V.G./Exc.^b^	32.5 (147)	67.4		11.6		16.0	
Poor or fair	67.5 (305)	53.4		7.9		16.7	
Number of Self-Reported Chronic Conditions			<0.01		0.61		0.20
0	26.1 (118)	78.8		10.2		13.7	
1	34.3 (155)	54.8		9.0		15.5	
≥2	39.6 (179)	46.9		8.4		19.2	
Depressed^c^			0.19		0.26		0.09
Yes	45.4 (205)	54.6		10.8		19.8	
No	54.7 (247)	60.7		7.7		13.8	
Health Care Access							
Cost-related access barrier^d^			0.81		0.86		0.03
Yes	45.1 (204)	57.4		9.4		20.7	
No	54.9 (248)	58.5		8.9		13.0	
Travel time to clinic in minutes			<0.01		0.69		0.01
0–29	39.6 (179)	69.8		8.9		12.4	
30–59	35.0 (158)	52.5		8.2		15.4	
≥60	25.4 (115)	47.0		10.5		24.4	

All percentages are row-percentages. ^§^Result of Pearson Chi-squared Test for binary predictors, result of Cochran-Armitage test of trend for variables with more than two levels;*One missing record; **Two missing records; °Result of Fisher’s Exact test for small cell sizes; ^a^Defined as not being able to read, frequently or always needing someone to help read papers from health center, frequently or always having problems understanding written medical instructions; ^b^Good/Very Good/Excellent; ^c^As measured by a score of 3 or more on the PHQ-2 questionnaire; ^d^“In the past year, did cost keep you from going to a clinic or hospital?”

In bivariate analyses (Table 1), there were significant (p < 0.05) positive associations between mobile phone ownership and: younger age (e.g. 96 % among respondents aged 18–29 versus 71% among respondents 65 and older); male sex (92 % versus 83 % of women); having high health literacy (93 % versus 72 % of individuals with low health literacy); more years of education (95 % of those individuals with more than 12 years of education versus 77 % among those individuals with no more than 6 years of education); and reporting no diagnosed NCDs (94 % among those without a diagnosed NCD, 87 % of individuals with 1 previously diagnosed NCD, and 81 % with at least two diagnosed NCDs). In multivariate analyses, younger age, male gender, and low health literacy remained significant, independent predictors (Table 3) of mobile phone ownership. Specifically, women had 0.41 times the odds of owning a mobile phone compared to men (95 % CI: 0.22–0.77) and respondents with low health literacy had 0.36 times the odds of owning a mobile phone as patients without these barriers (95 % CI: 0.20–0.64), after adjusting for covariates. Individuals aged 30–49 and individuals aged 50–64 had 2.4 and 2.5 times the adjusted odds, respectively, of owning a mobile phone (95 % CI: 1.10–5.21; 1.32–4.68, respectively) than those 65 and older (results not shown).

**Table 3:** Phone Ownership: Results of Multivariate Analysis (N = 559)

	Owns Mobile Phone		Owns Smartphone*	
	OR (95 % CI)	*p*-value	OR (95 % CI)	*p*-value
Demographics				
Age		0.02		0.03
18–29	Ref		Ref	
30–49	0.57 (0.12, 2.72)		0.64 (0.30, 1.37)	
50–64	0.60 (0.12, 2.85)		0.30 (0.13, 0.72)	
65+	0.24 (0.05, 1.18)		0.31 (0.11, 0.90)	
Gender		0.01		0.40
Male	Ref		Ref	
Female	0.41 (0.22, 0.77)		1.29 (0.71, 2.34)	
Indigenous language used at home		0.64		0.64
Yes	0.87 (0.50, 1.54)		0.85 (0.42, 1.70)	
No	Ref		Ref	
Health Literacy^a^		<0.01		0.04
Low	0.36 (0.20, 0.64)		0.40 (0.17, 0.94)	
High	Ref		Ref	
Education in years		0.28		<0.01
≤6	Ref		Ref	
7–12	1.18 (0.64, 2.17)		1.00 (0.43, 2.35)	
>12	2.10 (0.85, 5.21)		2.90 (1.25, 6.71)	
Health Status				
Self-Reported Health		0.63		0.09
G./V.G./Exc.^b^	Ref		Ref	
Poor or fair	1.15 (0.65, 2.05)		0.62 (0.35, 1.09)	
Number of Self-Reported Chronic Conditions		0.21		0.61
0	Ref		Ref	
1	0.47 (0.19, 1.17)		1.28 (0.64, 2.54)	
≥2	0.46 (0.19, 1.12)		1.47 (0.68, 3.19)	
Depressed^c^		0.71		0.10
Yes	1.11 (0.64, 1.93)		1.60 (0.92, 2.80)	
No	Ref		Ref	
Health Care Access				
Cost-related access barrier^d^		0.84		0.10
Yes	0.94 (0.55, 1.63)		0.61 (0.34, 1.10)	
No	Ref		Ref	
Travel time to clinic in minutes		0.57		0.29
0–29	Ref		Ref	
30–59	1.41 (0.74, 2.66)		0.75 (0.40, 1.39)	
≥60	1.14 (0.58, 2.21)		0.56 (0.26, 1.19)	

*Reference group included those that did not have a smart phone or had no mobile phone at all. ^a^Defined as not being able to read, frequently or always needing someone to help read papers from health center, frequently or always having problems understanding written medical instructions; ^b^Good/Very Good/Excellent; ^c^As measured by a score of 3 or more on the PHQ-2 questionnaire; ^d^“In the past year, did cost keep you from going to a clinic or hospital?”

In bivariate analyses (Table 1), smartphone ownership was markedly more common among individuals <30 years of age (32 %) than among those who were ages 50–64 (8 %) or ≥ 65 (6 %). Smartphone ownership was also more common among respondents who reported that they did not speak an indigenous language at home (15 % versus 8 % among indigenous language speakers), and among respondents with >12 years of education (26 %) versus <6 years (6 %). A larger proportion of respondents classified as having high health literacy owned a smartphone (16 %) than those classified as having low health literacy (5 %). In the multivariate model (Table 3), younger age, more years of education, and a high level of health literacy remained significant and positive independent predictors of owning a smartphone versus not owning a smartphone or mobile phone at all. The adjusted odds of owning a smartphone for individuals aged 65 and older were 0.31 times that of individuals aged 18–29 (95 % CI: 0.11–0.90). Those classified as having low health literacy had 0.40 times the adjusted odds of owning a smartphone when compared to those classified as being of high health literacy (95 % CI: 0.17–0.94). Compared to individuals with no more than 6 years of education, the adjusted odds of owning a smartphone were approximately 3 times greater (95 % CI: 1.25–6.71) among respondents with >12 years of education.

### Text messaging among respondents with a mobile phone

Text messaging was reported by the majority of all mobile phone owners, including among subgroups of respondents who had limited education, older age, and other indicators of socioeconomic vulnerability (Table 2). Fifty-four percent of the sample reported sending a text message at least once a week while 57 % received a text message once a week. When combined, sending or receiving text messages at least once a week was more common among younger respondents (94 % of those aged 18–29) than among older adults (31 % of those aged ≥ 65). Regularly sending and receiving text messages was also less common among respondents who reported speaking an indigenous language in their home (44 % versus 64 % among non-indigenous language speakers); those with low health literacy (38 % versus 65 % among patients without low health literacy); and those with fewer years of education (30 % among those with no more than 6 years of education versus 57 % and 82 % for those with 7–12 and > 12 years of education, respectively). Additionally in bivariate analyses, regular texting was less common among those who reported poor or fair perceived health, those with at least one NCD, and those with longer travel times to the clinic (Table 2).

Younger age, more years of education, fewer NCDs, and shorter travel time to the health clinic all remained significant positive predictors of texting at least once per week, after adjusting for covariates (Table 4). For example, when compared to individuals with no more than 6 years of education, those with more than 12 years of education had almost 6 times the adjusted odds of sending or receiving a text message at least once a week (95 % CI: 2.84, 10.99). Conversely, those traveling at least 60 min to the health clinic had 0.50 times the odds of sending or receiving text messages at least once per week as those traveling less than 30 min (95 % CI: 0.27, 0.90).

**Table 4:** Texting Among Mobile Phone Owners: Results of Multivariate Analysis (N = 452)

	Sends or receives at least once a week	
	OR (95 % CI)	*p*-value
Demographics		
Age		<0.01
18–29	Ref	
30–49	0.30 (0.08, 1.07)	
50–64	0.09 (0.03, 0.31)	
65+	0.07 (0.02, 0.25)	
Gender		0.37
Male	Ref	
Female	1.25 (0.77, 2.01)	
Indigenous language used at home		0.71
Yes	0.91 (0.54, 1.52)	
No	Ref	
Health Literacy^a^		0.14
Low	0.65 (0.37, 1.15)	
High	Ref	
Education in years		<0.01
≤6	Ref	
7–12	1.94 (1.09, 3.45)	
>12	5.59 (2.84, 10.99)	
Health Status		
Self-Reported Health		0.08
G./V.G./Exc.^b^	Ref	
Poor or fair	0.63 (0.38, 1.06)	
Number of Self-Reported Chronic Conditions		0.03
0	Ref	
1	0.41 (0.21, 0.79)	
≥2	0.59 (0.30, 1.14)	
Depressed^c^		0.24
Yes	0.75 (0.47, 1.21)	
No	Ref	
Health Care Access		
Cost-related access barrier^d^		0.29
Yes	1.30 (0.80, 2.12)	
No	Ref	
Travel time to clinic in minutes		0.04
0–29	Ref	
30–59	0.58 (0.34, 0.99)	
≥60	0.50 (0.27, 0.90)	

^a^Defined as not being able to read, frequently or always needing someone to help read papers from health center, frequently or always having problems understanding written medical instructions; ^b^Good/Very Good/Excellent; ^c^As measured by a score of 3 or more on the PHQ-2 questionnaire; ^d^“In the past year, did cost keep you from going to a clinic or hospital?”

### Barriers to mobile phone use

In bivariate analyses (Table 2), reporting frequent problems accessing a mobile signal was significantly more common among those with more education (e.g. 15 % among respondents with more than 12 years of education versus 7 % among those no more than 6 years). Lacking credit to make a call was significantly more common younger respondents (e.g. 33 % among those younger than 30 vs. 6 % among those at least 65 years old). Lacking phone credit also was more common among respondents reporting a cost-related access barrier (21 % versus 13 % among those not reporting this barrier) and among those with longer travel times to the clinic (e.g. 24 % among those traveling at least one hour versus 12 % among those traveling less than 30 min).

## Discussion

In this large survey of NCD patients recruited from outpatient clinics at four hospitals in Bolivia, we found that despite many patients’ socioeconomic risk factors, most owned a mobile phone and many used text messaging. While phone ownership was less common in certain sub-groups of the population, such as older adults or individuals with little or no formal education, even in these groups the majority of respondents were active mobile phone users. For instance, more than 70 % of respondents aged 65 years or older and 77 % of those with no more than 6 years of education owned a mobile phone. These findings suggest that even among some of the most vulnerable patients in one of the poorest countries of Latin America, mobile phone-based health interventions may be feasible.

The current study also highlights the potential reach of text messaging interventions in resource-constrained environments. More than half (58 %) of mobile phone owners reported that they send or receive text messages at least once a week. This is encouraging, because a large number of studies have shown that text messaging interventions can improve self-care behaviors and physiologic outcomes of NCD care – including in LMICs [6, 15, 34–37]. Texting was less common among older adults. However, given the rapid increase of text message utilization in the developing world [38] coupled with the positive results of studies using mHealth as a tool for behavior change across all age groups [6, 39], the feasibility of mHealth interventions will likely continue to increase across the age span.

Despite these positive messages, the current study does provide a cautionary note about focusing research and development of mHealth tools exclusively on smartphones and other Internet-enabled devices. Even in the youngest age group (ages 18–29) where smartphone use was most common, only one-third of respondents owned smartphones. While smartphone use likely will become more common in the future [40], interventions utilizing such phones will continue to have limited reach in the next decade at least. Nonetheless, since a quarter of respondents who had education beyond high school owned a smartphone, it may be possible to use these devices in mHealth interventions to offer training and support to health workers. More research will be needed to understand the extent to which smartphone owners in these settings are able and willing to use them to access health information through the Internet.

Overall, we found that relatively few mobile phone owners faced barriers to mobile phone use, with only 9 % reporting frequently or always lacking a mobile signal and 17 % frequently or always lacking mobile phone credit. Fortunately, in many areas of Bolivia, and Latin America in general, a person can receive a mobile phone call free of charge without a monthly subscription to a mobile phone plan or without having any phone credit [8], minimizing this cost-related barrier to one-way mobile phone-delivered interventions. Nevertheless, some of the same barriers to accessing traditional health care services also may serve as barriers to effective use of mHealth interventions. For example, older adults, females, and those with low health literacy were less likely to own a mobile phone, and older age, speaking an indigenous language at home, and lack of formal education were negatively associated with text messaging among mobile phone owners. These data also indicate that details such as access to “minutes” for making outbound calls are important and may vary in important ways across groups defined by socioeconomic risk factors. This pattern of effects is similar to the well-studied gap in access to health information in high-income countries such as the United States [41].

Strengths of the current study include the large sample size, the detailed questions about use of mobile phones, the fact that patients were recruited in four hospitals, and that it provides some of the most detailed information currently available on mobile phone access among NCD patients in the Andean region. However, the study also has certain limitations. Due to the cross-sectional design, the study provides limited insight into trends in this rapidly-changing field. For example, smartphone use was more common among younger patients, but it is unknown how rapidly smartphone penetration will increase across the different age groups of this population. Repeated cross-sectional studies will be needed to determine the rate at which this and other aspects of the mHealth landscape are changing in Bolivia and other similar LMICs. Our sample is also subject to selection bias since those individuals recruited at the hospitals may be different than individuals who do not seek formal medical care. Community-wide sampling, particularly in rural areas, may provide different estimates. Lastly, Bolivia is a culturally, linguistically, and geographically diverse country. Future mHealth research should target other regions of the country where patterns of ownership and utilization of mobile phones may be different.

## Conclusion

These data indicate that mobile phone ownership is common among NCD patients in the primary care setting in Bolivia, including among patients with limited education and other socioeconomic risk factors for poor health outcomes. Patients reported few problems with connectivity and most with a mobile phone reported frequent use of text messaging. These findings suggest that mHealth interventions that depend on NCD patients’ mobile phone ownership, including text messaging interventions, are feasible in this region and may reach the majority of patients in key subgroups, such as those who are older, those who from racial/ethnic minority groups, and those who have limited education.
